# Recent Advances in Chemical Vapor Deposition of Hexagonal Boron Nitride on Insulating Substrates

**DOI:** 10.3390/nano15141059

**Published:** 2025-07-08

**Authors:** Hua Xu, Kai Li, Zuoquan Tan, Jiaqi Jia, Le Wang, Shanshan Chen

**Affiliations:** 1 Beijing Key Laboratory of Optoelectronic Functional Materials and Micro-nano Devices, School of Physics, Renmin University of China, Beijing 100872, China; xuh2020@ruc.edu.cn (H.X.); likai0329@ruc.edu.cn (K.L.); 2017201149@ruc.edu.cn (Z.T.); jjq2022102228@ruc.edu.cn (J.J.); le.wang@ruc.edu.cn (L.W.); 2 Key Laboratory of Quantum State Construction and Manipulation (Ministry of Education), Renmin University of China, Beijing 100872, China

**Keywords:** h-BN, CVD, insulating substrates, direct growth, 2D materials

## Abstract

Direct chemical vapor deposition (CVD) growth of hexagonal boron nitride (h-BN) on insulating substrates offers a promising pathway to circumvent transfer-induced defects and enhance device integration. This comprehensive review systematically evaluates recent advances in CVD techniques for h-BN synthesis on insulating substrates, including metal–organic CVD (MOCVD), low-pressure CVD (LPCVD), atmospheric-pressure CVD (APCVD), and plasma-enhanced CVD (PECVD). Key challenges, including precursor selection, high-temperature processing, achieving single-crystalline films, and maintaining phase purity, are critically analyzed. Special emphasis is placed on comparative performance metrics across different growth methodologies. Furthermore, crucial research directions for future development in this field are outlined. This review aims to serve as a reference for advancing h-BN synthesis toward practical applications in next-generation electronic and optoelectronic devices.

## 1. Introduction

Hexagonal boron nitride (h-BN) is a wide-bandgap semiconductor material featuring a honeycomb-like layered structure composed of alternating boron and nitrogen atoms arranged in a planar hexagonal lattice with sp^2^ hybridization. The in-plane lattice parameter measures 2.5 Å, while the out-of-plane interlayer spacing is 6.6 Å [1,2]. h-BN exhibits an atomically flat surface with minimal dangling bonds and charge traps, making it an ideal candidate for gate dielectrics and substrate applications [3,4]. With a bandgap of 5.97 eV and a high absorption coefficient (~10^5^ cm^−1^) in the deep ultraviolet region [5,6], h-BN finds extensive applications in deep ultraviolet light-emitting devices and photodetectors [7,8]. Furthermore, its high thermal conductivity, excellent electrical insulating properties, and exceptional chemical stability enable the use of h-BN in diverse applications such as thermal management, dielectric materials, and corrosion-resistant coatings [9,10,11].

Chemical vapor deposition (CVD) has emerged as a predominant method for synthesizing two-dimensional materials (2DMs), where gaseous precursors decompose at high temperatures to form reactive species that deposit on substrates. This method is favored for its scalability and cost-effectiveness in producing large-area, high-quality thin films. However, the choice of substrate critically affects crystal quality, thickness uniformity, and overall film morphology. Most studies on CVD-grown h-BN have focused on metallic substrates, including copper (Cu) [12,13], nickel (Ni) [14,15], platinum (Pt) [16,17], iron (Fe) [18,19], and binary metal alloy [20,21], owing to their catalytic properties that promote more controllable growth. However, metal-based growth requires an additional transfer step, which remains a major challenge in 2DM fabrication. The transfer process introduces structural damages, chemical contaminations, and polymer residues, ultimately degrading material quality and impairing device performance [22,23]. To address these limitations, direct growth techniques on insulating substrates such as Si-based wafers and sapphire have been developed. Direct growth eliminates transfer-related defects, improves interface stability, and enhances compatibility with semiconductor manufacturing, laying a foundation for h-BN’s integration into electronic and optoelectronic applications.

This review highlights recent advancements in the CVD growth of h-BN on insulating substrates, covering various techniques such as metal–organic CVD (MOCVD), low-pressure CVD (LPCVD), atmospheric-pressure CVD (APCVD), and plasma-enhanced CVD (PECVD). Critical parameters influencing h-BN growth on insulating substrates are discussed, including precursor sources, growth substrates, temperature, and other process-specific factors. Furthermore, this review evaluates the advantages and limitations of various growth techniques and provides perspectives for future research on high-quality h-BN synthesis for advanced applications.

## 2. Structure of Boron Nitride

**Figure 1:**
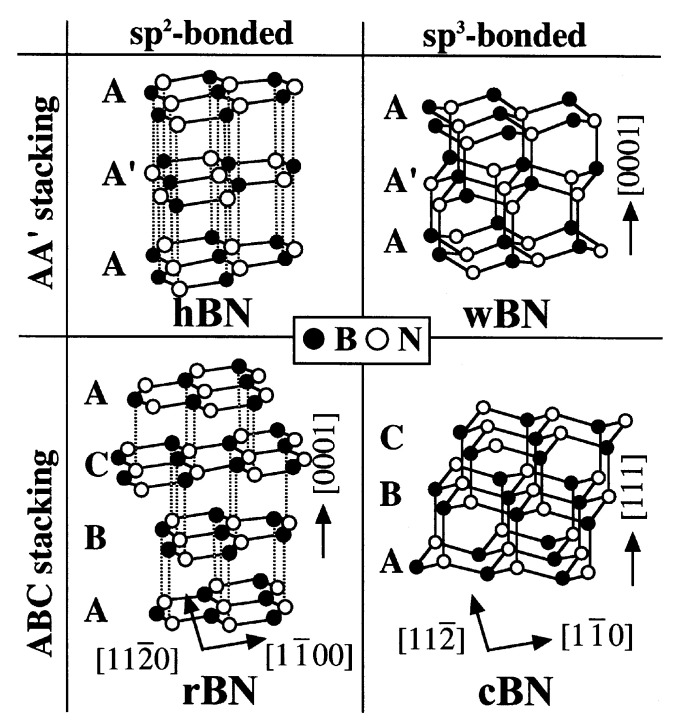
Atomic structure of h-BN, r-BN, w-BN, and c-BN [25]. Reproduced with permission [25]. Copyright 2001, Taylor and Francis.

Boron nitride (BN) is a quintessential III-V covalent compound. It can be classified into sp^2^-hybridized and sp^3^-hybridized structures based on the type of orbital hybridization [24]. As shown in Figure 1, the sp^2^-hybridized forms primarily consist of h-BN and rhombohedral boron nitride (r-BN) [25]. Among these two, h-BN is the most extensively researched two-dimensional material, characterized by a graphite-like layered stacking structure. Within each layer, B–N atoms are interconnected by robust covalent bonds, while the interactions between layers are governed by van der Waals forces. This unique arrangement imparts excellent thermal stability and electrical insulation properties. The interlayer stacking of h-BN can be categorized into two configurations: AA and AA’. In AA stacking, atoms in adjacent layers are perfectly aligned in the vertical direction. AA’ stacking, recognized as the most stable hexagonal configuration, features alternating B and N atoms between neighboring layers. Conversely, r-BN is an sp^2^-bound layered structure with layers arranged in an A-B-C stacking pattern, similar to rhombohedral graphite [26]. r-BN is a metastable phase and has been observed to transform into c-BN under high pressure (>8 GPa) at room temperature due to the similar stacking sequence [27].

In contrast, sp^3^-hybridized BN forms a three-dimensional network in which each B and An atom forms strong covalent bonds through tetrahedral coordination, resulting in a structure resembling that of diamond. The most notable phase of this type is cubic boron nitride (c-BN), which belongs to the cubic crystal system and possesses hardness comparable to that of diamond [27]. Another, less common form is wurtzite boron nitride (w-BN) [28]. While w-BN also exhibits high hardness, its thermodynamic stability is somewhat inferior to that of c-BN [29]. Overall, sp^3^-hybridized BN belongs to the category of conventional three-dimensional covalent crystals with remarkable mechanical properties, making it particularly well-suited for structural applications in extreme conditions.

## 3. Synthesis of h-BN via CVD

CVD is a widely used method for synthesizing high-quality h-BN thin films. As a bottom-up synthesis technique, it provides precise control over the crystallinity and thickness of the film [30]. Currently, CVD growth of h-BN mainly relies on metal-catalyzed substrates. These substrates can be divided into surface deposition and segregation mechanisms, depending on their solubility for boron and nitrogen precursors. Copper and platinum act as surface deposition catalysts [20,31,32]. Due to their low solubility for boron and nitrogen, h-BN growth occurs through surface-mediated reactions. In contrast, Ni, Fe-B, and Fe-Ni alloys rely on the segregation mechanism [14,33,34]. During cooling, dissolved boron and nitrogen separate from the metal and precipitate onto the surface. This process allows for thickness control through thermodynamic regulation.

Growing h-BN on insulating substrates through CVD follows a different mechanism. The process involves precursor decomposition, nucleation control, and interface interactions [2,35]. At high temperatures, gaseous precursors break down, releasing reactive boron and nitrogen species. These species diffuse onto the substrate surface, initiating nucleation and film formation. Unlike metal substrates, insulating materials such as Si and sapphire do not provide catalytic activity. As a result, the growth process depends on surface functionalization, interface engineering, and gas-phase kinetics control [30]. Controlling precursor transport and reaction rates is also crucial to produce uniform, high-quality thin films.

This section critically evaluates recent advances in the CVD synthesis of h-BN on dielectric substrates, beginning with precursor chemistry (boron/nitrogen sources) and following with a systematic comparison of MOCVD, LPCVD, APCVD, and PECVD methodologies.

### 3.1. B/N Precursors in CVD Synthesis of h-BN

The precursors for growing h-BN on insulating substrates via CVD can be classified into single B/N compounds and dual B/N compounds. Single B/N reactant contains both boron and nitrogen elements within their structure. Upon heating, these compounds decompose, releasing the necessary B and N species, which then deposit onto the insulating substrate to form h-BN. In contrast, binary B/N compounds consist of a boron-containing organic or inorganic compound and a nitrogen source, typically ammonia (NH_3_) or nitrogen (N_2_), with h-BN growth being regulated by adjusting the V/III ratio.

Table 1 systematically catalogs the B/N precursors currently utilized in CVD processes for h-BN growth [36,37,38,39,40,41,42,43]. Diborane (B_2_H_6_) and NH_3_ were among the early precursors used in the CVD growth of BN [44]. Diborane is a colorless gas at room temperature and atmospheric pressure, characterized by its extremely high chemical reactivity. It can form explosive mixtures with air upon mixing and is also highly toxic. Although diborane serves as an excellent carbon-free boron source for h-BN growth, its explosive characteristics and high toxicity require stringent safety protocols in laboratory applications, consequently elevating experimental complexity [45].

Most MOCVD research tends to utilize alternatives such as triethylborane (TEB) or trimethylborane (TMB) instead [46,47]. TEB is a liquid at room temperature, while TMB is gaseous, making them suitable for different deposition conditions. These organic compounds decompose at high temperatures to generate elemental boron, which reacts with NH_3_ to form h-BN. However, a key limitation of these alkane-based precursors is their incomplete decomposition at low temperatures, which can lead to inefficient boron incorporation. At higher temperatures, though decomposition improves, they tend to introduce carbon impurities into the h-BN films, compromising crystallinity.

Moreover, there are several types of boron source precursors available, including trimethoxy borate (TMOB, (B(OCH_3_)_3_) [48], borane-dimethylamine (DMAB, BH_3_NH(CH_3_)_2_) [49,50], boron trichloride (BCl_3_) [51], and boron trioxide (B_2_O_3_) [52]. TMOB and DMAB are known for their low decomposition temperatures and ability to undergo complete thermal decomposition, making them well-suited for the CVD process. However, since these two precursors contain carbon elements, it is challenging to avoid introducing impurities during their use, which can lead to various structural defects. BCl_3_ is particularly sensitive during the reaction process. It generates hydrochloric acid (HCl) gas when reacting with hydrogen sources, which can not only interfere with h-BN growth but also potentially corrode quartz tubes, affecting the stability of the equipment [53]. Therefore, it is essential to maintain a dry and sealed growth system when using BCl_3_. Inert gases such as N_2_ or argon (Ar) are usually employed to protect the process and ensure a stable reaction environment. Boron trioxide (B_2_O_3_) is a widely used boron oxide, known for its excellent chemical stability. It is commonly used to produce borates and glassy materials. When heated to about 450 °C, B_2_O_3_ decomposes to release boron ions, which play a role in forming h-BN [52]. However, B_2_O_3_ is hygroscopic and reacts with moisture in the air to form boric acid. Therefore, controlling the humidity during the reaction is crucial for preventing material degradation and ensuring stable reaction conditions.

In recent years, single boron–nitrogen compounds, represented by borazine (B_3_N_3_H_6_) [37,54] and ammonia borane (NH_3_-BH_3_) [55], have gained increasing attention in h-BN growth. Borazine, often called “inorganic benzene”, is a colorless liquid at room temperature with high chemical stability. Its molecular structure consists of alternating boron and nitrogen atoms, forming a conjugated system that remains thermally and chemically stable even at elevated temperatures. Borazine is highly flammable and can ignite when exposed to open flames or hot surfaces, releasing toxic gases such as NH_3_ and B_2_H_6_. To ensure safety, borazine must be handled within a sealed system, equipped with proper exhaust treatment facilities, like a scrubber, to manage and neutralize hazardous emissions [56].

Ammonia borane (AB), on the other hand, is a solid-state molecular complex, characterized by strong dative bonding between the boron and nitrogen atoms [57,58]. Different from borazine, AB is considerably safer to handle and store, making it an attractive precursor for h-BN deposition in various CVD techniques. Both borazine and AB are free from impurity elements and decompose at relatively low temperatures. Compared to borazine and other boron-containing precursors, AB has lower toxicity and reduced volatility, which enhances handling safety and simplifies transport. Due to its relatively low decomposition temperature and the absence of carbon-containing byproducts, AB is well-suited for direct h-BN growth on both catalytic and non-catalytic substrates. Moreover, its intrinsic B:N = 1:1 ratio ensures elemental balance during thermal decomposition, positioning it as an excellent candidate for further application in h-BN synthesis [59].

While boron sources exhibit significant diversity in h-BN synthesis, nitrogen sources are comparatively limited. Among them, NH_3_ serves as the predominant gaseous nitrogen source for h-BN growth via conventional CVD [60], while N_2_ is primarily utilized in the PECVD technique [61], where plasma technology effectively reduces its dissociation temperature. Recently, there have been attempts to use ammonium carbonate [(NH_4_)_2_CO_3_] as a new solid N precursor, which can be decomposed and release module gas when heated at 60 °C [52,62]. However, the substances it releases contain CO_2_, which may introduce carbon defects into the growth of h-BN.

**Table 1:** B/N Precursors applied in CVD Synthesis of h-BN.

	Precursors		Physical State	T_dec_ (°C)	Byproduct	Safety	Technique
**Single** **Reactant**	Ammonia borane (AB, NH_3_-BH_3_)		Solid	67 [36]	-	-	LPCVD, APCVD, PECVD
	Borazine (B_3_N_3_H_6_)		Liquid	375 [37]	-	Flammable, toxic	MOCVD, PECVD
**Dual Reactants**	Diborane (B_2_H_6_)	NH_3_ *	Gas	200 [38]	-	Flammable, toxic	MOCVD, LPCVD
	Trimethylboron (TMB, B(CH_3_)_3_)	NH_3_ *	Gas	300 [39]	Carbon impurities	Flammable, toxic	MOCVD, LPCVD
	Triethylboron (TEB, (B(CH_3_CH_2_)_3_)	NH_3_ *	Liquid	400 [39]	Carbon impurities	Flammable, toxic	MOCVD, LPCVD
	Trimethoxy borate (TMOB, B(OCH_3_)_3_)	N_2_ *	Liquid	700 [40]	Carbon impurities	Flammable, toxic	PECVD
	Dimethylamine-borane (DMAB, BH_3_NH(CH_3_)_2_)	N_2_ *	Solid	120 [41]	Carbon impurities	Flammable, toxic	PECVD
	Boron trichloride (BCl_3_)	NH_3_ *, N_2_ *	Gas	477 [42]	HCl	Highly corrosive	LPCVD, PECVD
	Boron trioxide (B_2_O_3_)	Ammonium carbonate, ((NH_4_)_2_CO_3_)	Solid	B_2_O_3_: 650 [43] (NH_4_)_2_CO_3_: 60 [62]	-	-	APCVD

* The decomposition temperature (T_dec_) of NH_3_ is 900 °C [60], while that of N_2_ is 1727 °C [61].

### 3.2. h-BN Growth via MOCVD

MOCVD is a thin-film growth technique that utilizes metal–organic compounds as precursors to deposit epitaxial layers via controlled chemical reactions on substrate surfaces. Renowned for its precise thickness control and superior crystallinity, MOCVD has emerged as the dominant method for synthesizing III-nitrides and a pioneering approach for h-BN fabrication. Typically, metal–organic precursors, such as TEB and TMB, decompose in the gas phase and react with NH_3_ on the heated substrate surface to form h-BN films. The growth kinetics are governed by precursor flow dynamics, nucleation mechanisms, and interfacial interactions. Recent advances highlight that optimizing the V/III ratio, engineering substrate surfaces, and fine-tuning deposition temperatures are critical for enhancing h-BN film quality.

Table 2 summarizes the development of MOCVD-grown h-BN, from early research to modern optimization techniques. The earliest use of MOCVD for h-BN synthesis dates back to 1968, when Rand and Roberts deposited amorphous and polycrystalline BN films on silicon, tantalum, and fused silica substrates using B_2_H_6_ and NH_3_ at 600~800 °C [44]. Recent research demonstrates that B_2_H_6_, as a carbon-free boron precursor, reduces carbon impurities and improves crystallinity in sapphire-grown h-BN films compared to metal oxide alternatives (e.g., TMB) [63]. Despite its effectiveness, B_2_H_6_ is rarely used in MOCVD h-BN synthesis due to safety concerns (high toxicity) [64,65]. Instead, metal oxide precursors dominate industrial and academic applications.

As a room-temperature liquid, TEB demonstrates reduced reactivity relative to TMB, offers enhanced storage stability, and serves as the dominant boron precursor for MOCVD-based h-BN growth [46,66,67,68,69,70,71,72,73,74,75]. By 1986, Nakamura et al. demonstrated that adjusting the precursor molar ratio of TEB and NH_3_ significantly improved film crystallinity. Deposited on sapphire at 950–1000 °C, the films achieved hexagonal structures with an optical bandgap of 5.90 eV [46]. By 2008, Kobayashi and Akasaka demonstrated epitaxial growth of (0001)-oriented h-BN films on sapphire at 1080 °C using a high V/III precursor ratio (above 1280), whereas a low V/III ratio (such as 210) resulted in the obtainment of turbostratic BN (t-BN) [66]. From 2014 to 2019, multiple research groups successively achieved the effective growth of polycrystalline h-BN on sapphire substrates using TEB as the boron precursor with ammonia [67,68,69,70,71,72,73,74]. The growth temperature window was established between 950 and 1350 °C, producing films with tunable thickness ranging from 1.5 nm to 70 nm. Notably, ultra-thin films with thicknesses of 1–2.5 μm were also successfully obtained [71]. Raman spectroscopy characterization revealed full width at half maximum (FWHM) values in the range of 25–45 cm^−1^, highlighting MOCVD’s potential for achieving high-quality epitaxial layers.

**Figure 2:**
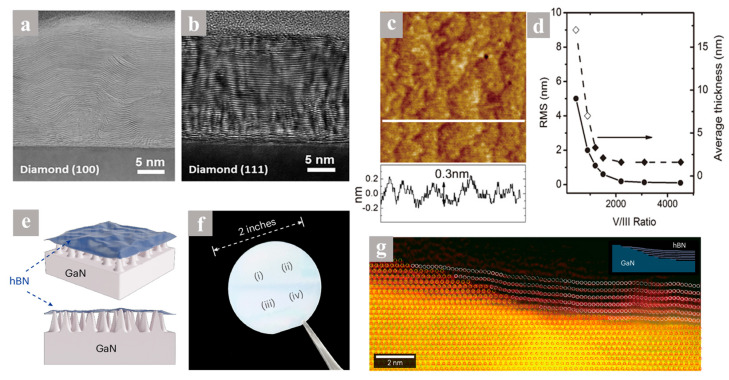
(**a**,**b**) Cross-sectional bright-field STEM image of h-BN on diamond (100) (**a**) and diamond (111) (**b**) substrates [76]. (**c**,**d**) AFM images of h-BN on sapphire deposited using V/III ratios of 4500 (**c**) and surface roughness and average thickness as a function of V/III ratio (**d**) [67]. (**e**–**g**) Schematic of the h-BN film on GaN nanoneedles grown by MOCVD (**e**), photograph of an as-grown two-inch h-BN film on a GaN/sapphire wafer (**f**), and low-magnification STEM of the nucleation of AA-h-BN from the step edge of the GaN substrate (**g**) [81]. (**a**,**b**) Reproduced with permission [76]. Copyright 2020, IOP Publishing. (**c**,**d**) Reproduced with permission [67]. Copyright 2014, ACS. (**e**–**g**) Reproduced with permission [81]. Copyright 2025, Springer Nature.

Besides regulation through the V/III precursor ratio, the selection of a proper substrate, the single crystallinity of the substrate, and its structural compatibility with h-BN as well as the annealing process are critical parameters for optimizing the crystalline quality of as-grown h-BN films. In 2020, Yang et al. successfully grew ordered h-BN films on diamond (111) substrates and disordered h-BN films on diamond (100) substrates at 1380 °C, which was attributed to the hexagonal symmetry of the diamond (111) surface (Figure 2a,b) [76]. Via a post-annealing process at 1600 °C, Lee et al. found that annealing h-BN films grown at 1050 °C exhibit enhanced crystallinity and homogeneity [75]. By using single-crystalline sapphire substrates with a 1° off-cut angle and performing pre-nitridation and annealing processes, Tokarczyk et al. reported the synthesis of a h-BN film with Raman FWHM decreased to 25 cm^−1^ and surface roughness reduced to 0.15 nm [77]. In 2013, Majety et al. synthesized h-BN on n-type 6H-SiC and achieved band-edge photo-luminescence at 5.5 eV, which opened up new substrates for the application of h-BN in high-performance optoelectronic devices [78].

Thickness control in MOCVD-grown h-BN films was achieved by modulating the growth mode. In 2014, Paduano et al. demonstrated a self-terminating growth mechanism on sapphire at 1050 °C, producing atomically smooth multilayer h-BN films with uniform thickness (Figure 2c) [67]. They found that using a high V/III precursor ratio under low-pressure conditions (20–300 Torr) shifted the growth mode from random 3D nucleation to self-terminating growth. This approach offers a stable process window, enabling precise control over the number of atomic layers in h-BN films.

Direct growth of h-BN on sapphire substrates triggers dual mismatch effects (lattice + thermal expansion), generating compressive stress at the interface and leading to wrinkle formation due to thermal gradient relaxation. To diminish the influence of the localized strain caused by the hetero-substrate, a buffer layer is typically introduced. By using a two-step process, self-limiting BN buffer layers grown at 800 °C were introduced to grow more uniform h-BN films at 1300 °C [78,79]. Aleksandra et al. further proposed a growth scheme that involves an intermediary BN buffer layer grown under self-limiting conditions (continuous flow) followed by the final growth of h-BN with flow modulated epitaxy [80]. The study shows that the buffer layer allows lowering the density of point-like defects and could effectively suppress the creation of amorphous BN (a-BN) at the sapphire/h-BN interface. The roughness of the h-BN epitaxy layer was reduced to 0.2 nm with the residual stress was found to induce lattice distortions of less than 1.5%. Besides BN homo-buffer layer, AlN buffer layer was also used to optimize the initial nuclei of BN seeds, which further formed the cap-shaped-like layer and eventually grow into highly (0001)-oriented 2D multilayer h-BN with a surface roughness of 0.25 nm [80]. Recent research indicates that the pre-introduction of a GaN buffer layer on the sapphire surface not only enhances lattice adaptation but also facilitates the epitaxial growth of AA-stacked h-BN by creating a Ga vicinal step structure as shown in Figure 2e–g [81]. In a high-temperature hydrogen atmosphere, the GaN buffer layer is etched to form a nanoneedle-like structure, which supports the h-BN film above, resulting in a suspended configuration. This effectively alleviates stress within the film and mitigates wrinkles and cracks that can arise from mismatches in the crystal lattice and thermal expansion. Experimental findings demonstrate that the sample grown with AA h-BN on a GaN substrate exhibits exceptionally high crystal quality and an optical nonlinear response, with the second harmonic generation (SHG) signal intensity exceeding that of AA’ h-BN of the same thickness by over tenfold. These results show that the buffer layer plays a crucial role in enhancing the uniformity and quality of h-BN films.

Doping plays a critical role in modulating the electronic properties of III-V compound semiconductors (e.g., GaN [82] and AlN [83]), enabling tailored carrier concentration and band engineering for optoelectronic and high-frequency applications. However, h-BN, despite its exceptional thermal conductivity and ultra-wide bandgap, remains understudied in doping research due to challenges in achieving stable substitutional doping and controlling defects during synthesis. In 2011, Dahal et al. achieved p-type doping of h-BN films by introducing biscyclopentadienyl-magnesium during the growth process [84]. These films, grown on sapphire substrates at 1200 °C, demonstrated a doping concentration of 1 × 10^19^ cm^−3^ and a p-type resistivity around 12 Ω·cm at 300 K. Compared to Mg-doped wurtzite AlN, h-BN epilayers have realized dramatic reductions in Mg acceptor energy level and P-type resistivity by about six to seven orders of magnitude.

Although metal–organic precursors such as TEB and TMB are ubiquitously employed in MOCVD-grown h-BN, their utilization presents a critical limitation: inevitable carbon contamination in epitaxial layers [46,63,65,73]. At high temperatures, TM-precursors decompose breaking down their organic groups and introducing carbon impurities into the h-BN films. These impurities can result in unwanted doping, negatively impacting the electronic and optical properties of the material. Additionally, carbon contamination can degrade interface quality, reducing film uniformity and crystallinity. To address this issue, researchers have explored alternative boron sources, such as diborane [63] and borazine [54], which have shown the potential to reduce carbon contamination while improving nucleation efficiency and overall film quality under optimized conditions.

**Table 2:** Growth of h-BN films on insulating substrates via MOCVD.

Precursor	Substrate	Growth Temperature (°C)	Raman FWMH (cm^−1^)	Thickness (nm)	Deposition Rate (nm/min)	Structure	Year
B_2_H_6_, NH_3_	Si, Ta, Fused Si	600–800	-	100–600	5–100	Below 800 °C: **a-BN**	1968 [44]
						800 °C: mixture of **h-BN** and **a-BN**	
	Sapphire	1300	24.5	3.2	0.32	**h-BN** film	2021 [64]
		1160–1400	-	41–123	0.6–2.1	Mixture of **h-BN** and **t-BN**	2023 [65]
B_2_H_6_, NH_3_ or TMB, NH_3_	Sapphire	1360	B_2_H_6_: 21.8	B_2_H_6_: 80	B_2_H_6_: 3.6	B_2_H_6_: **h-BN** film	2020 [63]
			TMB: 42.7	TMB: 40	TMB: 1.6	TMB: **t-BN** film	
TEB, NH_3_	Sapphire	950–1000	-	-	16	**h-BN** film	1986 [46]
		1080	-	300	V/III ratio of 1280: 0.5	V/III ratio of 1280: **h-BN** film	2008 [66]
					V/III of ratio 210: 2	V/III of ratio 210: **t-BN** film	
		1050	26–30	V/III ratio of 3100: 1.6	0.05	**h-BN** film	2014 [67]
				V/III ratio of 450: 17			
		950–1100	25–32	1.6	0.3		2016 [68]
		1100	25–30	1.5	-		2016 [69]
		1280	45	3–60	0.25		2016 [70]
		1280	33.2	1.0 μm–2.5 μm	0.25		2017 [71]
		1330	-	70	1.2		2017 [72]
		1350	27	20	0.04		2018 [73]
		1280	41	30–60	0.25		2019 [74]
		1050	28.84	3.07	0.08		2019 [75]
		(Post-annealed at 1700)					
	Diamond (100), (111)	1380	-	7.4	-	Single crystalline **h-BN** film	2020 [76]
	Sapphire (1° off-cut)	1280–1300	29.5	21	0.35	Mixture of **h-BN** and **t-BN**	2023 [77]
	AlN buffer layer/Sapphire	1300	-	1 μm	-	**h-BN** film	2011 [79]
	BN buffer layer/6H-SiC	1300	-	500	-	Single crystalline **h-BN** film	2013 [78]
	AlN buffer layer/Sapphire	1380	-	40	25	h-BN [(0001] || AlN [0001]	2020 [80]
						h-BN [(10$\overset{-}{1}$0] || AlN [11$\overset{-}{2}$0]	
	GaN buffer layer/Sapphire	1050	-	2.5	0.06	AA stacking h-BN Single crystallinel film	2025 [81]
TEB, NH_3_, biscyclopentadienyl-magnesium	AlN buffer layer/Sapphire	1300	-	-	-	Mg-doped **h-BN** film	2011 [84]
TMB, NH_3_	Sapphire	1400	-	50 μm	-	Single crystalline **h-BN** film	2018 [47]
Borazine	Sapphire	1500	29.5	30	0.17	Single crystalline **h-BN** film	2021 [54]

Unless otherwise specified, the ‘h-BN film’ referred to in the table is polycrystalline.

### 3.3. h-BN Growth via LPCVD

LPCVD is widely used for growing high-quality thin films due to its low-pressure environment, longer precursor residence time, and stable gas-phase transport. The reduced pressure minimizes gas-phase collisions, enabling precise layer-by-layer deposition. LPCVD provides superior control over film thickness, enhanced crystallinity, and excellent uniformity across large areas [85]. These advantages make LPCVD an excellent choice for synthesizing two-dimensional materials, where uniformity and crystallinity are critical. Table 3 summarizes recent advances in LPCVD-grown h-BN films, highlighting optimized precursor supply, substrate modifications, and growth temperature control for improved film quality.

Early researchers initially adopted conventional MOCVD precursors TEB and NH_3_ for the LPCVD growth of h-BN [86,87,88,89,90,91]. However, significant differences emerged between MOCVD and LPCVD systems in the phase control of h-BN synthesis. Unlike MOCVD’s relative ease in h-BN synthesis, LPCVD processes using TEB demonstrated strict temperature sensitivity for pure-phase h-BN formation on insulating substrates. Both suboptimal low and excessive high temperatures predominantly yielded t-BN or r-BN, with a narrow optimal window for h-BN crystallization. Jin et al. achieved multilayer t-BN growth on Si (100) substrates at 850–1000 °C through TEB pyrolysis [86]. Additionally, Ahmed et al. successfully grew h-BN on Si (111) substrates at 1350 °C using a cold wall CVD system [90]. Nitridation sapphire substrates to create AlN buffer layers were further implemented to achieve the phase control of as-grown BN. Chubarov et al. report the growth of h-BN on AlN/sapphire templates at 1200–1500 °C [88]. They found that h-BN could change to r-BN when the thickness is up to 4 nm at 1500 °C. Ahmed et al. further studied nitridation temperature (T_N_) modulation of buffer layers, discovering that amorphous AlN_x_O_1−x_ interlayers significantly enhanced h-BN growth kinetics [89]. Building on this, Sharma et al. achieved BN crystal phase engineering through substrate orientation control. Using AlN-buffered sapphire with specific crystallographic orientations, r-BN was found to form on Al_2_O_3_ (11$\overset{-}{2}$0) and Al_2_O_3_ (0001) [91]. In brief, the synthesis of h-BN using TEB as a precursor in LPCVD systems demands exceptionally precise control over growth parameters. The inherent challenges stem from TEB’s complex decomposition kinetics, boron-rich stoichiometry, and propensity for forming metastable phases like t-BN or r-BN.

**Figure 3:**
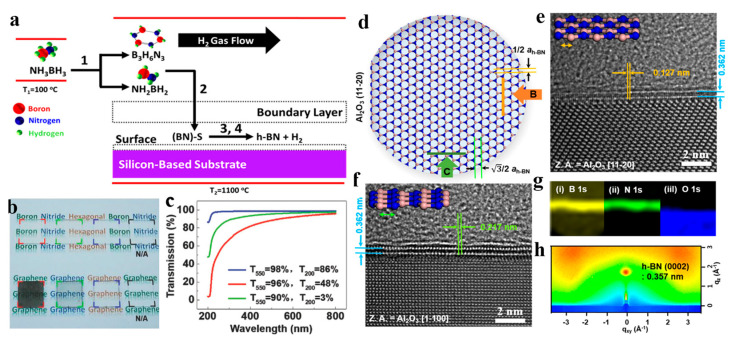
(**a**) Schematic of h-BN growth on Si-based substrate [94]. (**b**,**c**) Photograph of h-BN/quartz and graphene/quartz samples with different thicknesses from monolayer to over 20 layers (**b**) and (**c**) the corresponding transmittance spectra of the samples marked in (**b**) [95]. (**d**) Schematic illustration of epitaxial-grown h-BN on a sapphire substrate [97]. (**e**,**f**) HR-TEM images of multilayer h-BN grown perpendicular to Al_2_O_3_ (11$\overset{-}{2}$0) (**e**) and parallel to Al_2_O_3_ (11$\overset{-}{2}$0) (**f**) [97]. (**g**,**h**) EF-TEM images for B, N, and O 1s and (**e**) GI-WAXD result of h-BN on a sapphire substrate [97]. (**a**) Reproduced with permission [94]. Copyright 2017, ACS. (**b**,**c**) Reproduced with permission [95]. Copyright 2018, Wiley. (**d**–**f**) Reproduced with permission [97]. Copyright 2016, ACS.

Solid-state AB has emerged as the predominant precursor for the LPCVD synthesis of h-BN, owing to its intrinsic advantages including the 1:1 stoichiometric B:N ratio ensuring defect-minimized growth, the safety for transport and storage, and its high purity eliminating unintended dopant incorporation [55,92,93,94,95,96,97,98,99]. Unlike TEB-based processes requiring stringent temperature control to avoid t-BN phases, AB enables direct h-BN crystallization with superior phase purity. As shown in Figure 3a, AB thermally decomposed to aminoborane (NH_2_BH_2_), B_3_H_6_N_3_, and H_2_ at 67–135 °C [94]. Then, these precursors’ molecules are absorbed on the substrate and dehydrogenated at 700–1100 °C to form h-BN. In 2014–2018, there have been multiple studies that have achieved thickness-tunable polycrystalline h-BN across diverse insulating substrates (Si, SiO_2_/Si, Si_3_N_4_, c-plane and r-plane sapphire) at 1000–1100 °C with AB decomposition rates optimized via carrier gas modulation [55,90,91,92,93]. Figure 3b,c present representative h-BN/quartz samples with thicknesses ranging from 1 to 20 atomic layers, along with their corresponding transmittance spectra [93].

Growth temperature serves as a pivotal determinant for the crystallinity of h-BN synthesized on insulating substrates with AB as the B/N precursor. Jang et al. successfully grew wafer-scale, multilayer h-BN at 1400 °C using AB on c-plane sapphire. A high-resolution transmission electron microscopy (HRTEM) study indicated a single rotational orientation with the AA′ stacking order (Figure 3d–g) [97]. Elevated temperatures promote c-axis-oriented h-BN growth while suppressing t-BN phase formation. In 2023–2024, Chen et al. demonstrated the controllable growth of high-crystallinity h-BN on c-plane and a-plane sapphire substrates under 1400 °C using an AB precursor [96,98]. This work highlighted the critical role of the growth temperature in achieving superior sample quality h-BN by LPCVD.

**Figure 4:**
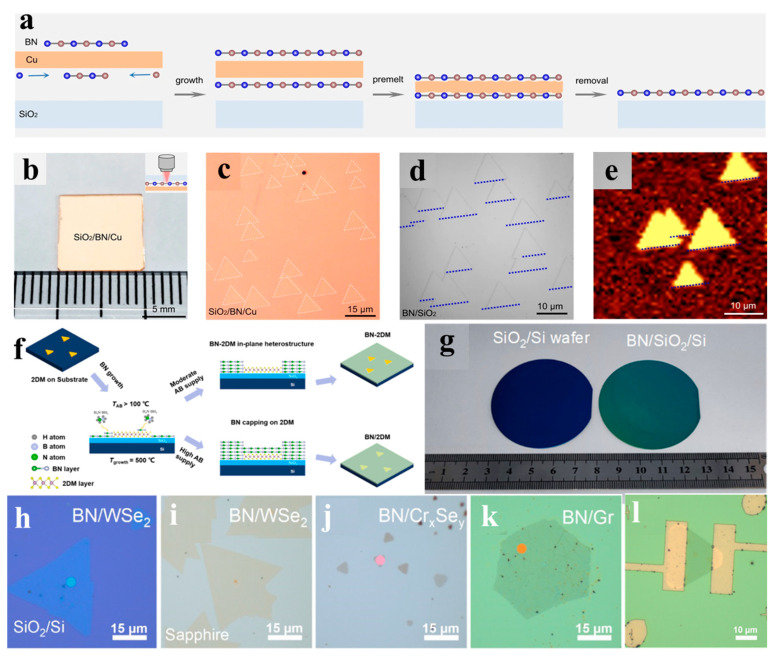
(**a**) Schematic diagrams of the growth process of h-BN via a Cu-assisted CVD strategy [100]. (**b**–**e**) Optical image of the SiO_2_/h-BN/Cu sandwiched structure (**b**), zoom-in optical image of SiO_2_/h-BN/Cu (**c**), optical image of as-grown h-BN islands on SiO_2_ after removing the Cu foil (**d**) and second harmonic generation intensity mapping of h-BN islands (**e**) [100]. (**f**,**g**) Schematic diagram of a-BN grown on 2DMs with different amounts of AB supply (**f**) and photograph of the wafer-scale fully covered a-BN film grown directly on a two-inch SiO_2_/Si wafer (**g**) [104]. (**h**–**l**) Optical images of WSe_2_/SiO_2_/Si (**h**), WSe_2_/Sapphire (**i**), Cr_x_Se_y_/mica (**j**) and graphene/SiO_2_/Si after a-BN capping (**k**) [104]. (**l**) Optical image of the WSe_2_ FET after a-BN capping [104]. (**a**–**e**) Reproduced with permission [100]. Copyright 2023, Springer Nature. (**f**–**l**) Reproduced with permission [104]. Copyright 2022, ACS.

While metallic substrates enable effective growth of monolayer h-BN via self-limiting mechanisms due to their high catalytic activity, achieving monolayer h-BN on insulating substrates remains a big challenge. To address this, Zeng et al. pioneered a Cu-assisted CVD strategy [100]. As depicted in Figure 4a–e, by introducing a Cu (110) foil on diverse insulating substrates (SiO_2_ (001), SrTiO_3_ (001), c-plane sapphire, and fused silica), they achieved single-crystalline h-BN monolayers on both sides of the Cu foil. Then, through an after-growth annealing process at 1087 °C, the Cu foil was melted and tightly stuck to the substrate. Finally, the Cu layer was etched in (NH_4_)_2_S_2_O_8_ solution, leaving purely insulating-supported h-BN without metallic residues [100].

Beyond AB, researchers have explored alternative gas-phase precursors for h-BN synthesis, such as B_2_H_6_/NH_3_ [51] and BCl_3_/NH_3_ [101,102,103], each offering distinct kinetic advantages. In 2021, Bansal et al. demonstrated a pulsed flow-modulated epitaxy (FME) approach using sequential injections of B_2_H_6_ and NH_3_, combined with optimized V/III ratios, enabling the growth of ultrathin h-BN films (~3 nm) with improved layer uniformity [51]. Similarly, Umehara et al. adopted BCl_3_/NH_3_ chemistry for polycrystalline h-BN growth on c-plane sapphire. They identified two critical control parameters to be the growth temperature and the gas flux ratio [101,102]. Independently, Xi Chen et al. applied BCl_3_/NH_3_ onto the Si (100) substrate. Their temperature-dependent analysis revealed a strong correlation between thermal energy and crystallinity. And the optimized growth temperature was 1200 °C for h-BN films on the silicon substrate with BCl_3_/NH_3_ precursors [103].

While 1000 °C is essential for producing high-quality h-BN, the development of a-BN at low temperatures has also gained significant interest due to its potential applications as a multifunctional encapsulation layer [104,105,106]. In 2022, Lu et al. demonstrated a precursor amount supply modulated technique to acquire a-BN at 500 °C via LPCVD [104]. As shown in Figure 4f,g, by precisely tuning the amount of the supply of AB on insulating substrates such as SiO_2_/Si, Al_2_O_3_, and quartz, they successfully produced tunable a-BN films with thicknesses ranging from 4 nm to 30 nm. Their study further revealed that a-BN can be directly synthesized on 2D material surfaces (e.g., graphene, WSe_2_, Cr_x_Se_y_), functioning as an ultrathin protective barrier to maintain structural stability under ambient exposure and laser irradiation (Figure 4h–k). This low-temperature growth technique can be integrated onto prefabricated FET devices, forming atomically clean and conformal BN/2D material interfaces, which significantly improves the stability and mobility of the devices (Figure 4l). In 2023, Lee et al. further reduced the growth temperature to 250 °C and confirmed the successful growth of a-BN by XPS [105]. They characterized the electrical properties of low-temperature-grown a-BN, revealing an ultralow dielectric constant of 1.25 at 1 MHz—a value comparable to that of air. The encapsulated devices created using this process further highlight the advantages of a-BN as a protective layer.

**Table 3:** Growth of h-BN films on insulating substrates via LPCVD & APCVD.

	Precursors	Substrates	T_dec_ (°C)	Growth Temperature (°C)	Raman FWMH (cm^−1^)	Thickness (nm)	Deposition Rate (nm/min)	Structure	Year
**LPCVD**	TEB, NH_3_	Si (100)	-	850–1100	-	-		**t-BN** film	1998 [86]
		Sapphire, AlN buffer layer/Sapphire	-	1500	31	400	1.67	Sapphire: **t-BN** AlN/Sapphire: **r-BN**	2011 [87]
		AlN buffer layer/Sapphire, 6H-SiC	-	AlN/Sapphire: 1200–1500 6H-SiC: 1600	-	-	0.33	AlN/Sapphire: **h-BN** at 1200 °C **r-BN** at 1500 °C 6H-SiC: **r-BN** at 1600 °C	2015 [88]
		Amorphous AlN_x_O_1−x_ buffer layer/Sapphire	-	1350	25	300	7.5	T_N_: Buffer layer nitridation temperature Lower T_N_: **h-BN** film Higher T_N_: **t-BN** film	2016 [89]
		Si (111)	-	1350	25	1.6 μm	27	**h-BN** film	2016 [90]
	TEB, NH_3_/TMB, NH_3_	AlN buffer layer/Sapphire (0001), (11$\overset{-}{2}$0), (1$\overset{-}{1}$02), (10$\overset{-}{1}$0)	-	TMB: 1400 TEB: 1500	-	TMB: 150–1000 nm TEB: 300 nm	2.5	**r-BN** [(11$\overset{-}{2}$0] || AlN [(11$\overset{-}{2}$0]|| Al_2_O_3_ [0001] **r-BN** [(11$\overset{-}{2}$0] || AlN [1$\overset{-}{1}$02]|| Al_2_O_3_ [0010] **a-BN** || Al_2_O_3_ (1$\overset{-}{1}$02) **a-BN** ||Al_2_O_3_ (10$\overset{-}{1}$0)	2022 [91]
	AB	Si (111) Sapphire	135	1000	-	25	2.5	**h-BN** film	2014 [55]
		Quartz, Si	100	1100	-	7	-		2015 [92]
		Quartz, SiO_2_/Si	100	1000	42–46	2–25	0.4–0.8		2015 [93]
		Si, Si_3_N_4_, SiO_2_	100	700–1100	-	5	0.1		2017 [94]
		Sapphire, SiO_2_	75–90	1100	-	monolayer~20 layers	-		2018 [95]
		Sapphire	130	1400	-	-	-	Single crystalline AA’ **h-BN** film	2016 [97]
		Sapphire	115	1400	37.94–39.24	-	-	High crystalline **h-BN** film	2023 [96]
		Sapphire (11$\overset{-}{2}$0)	115	1400	<30	3–35	0.58	[1100]**h-BN**//Al_2_O_3_[11$\overset{-}{2}$0]	2024 [98]
		SiO_2_, Sapphire, Mica, MoS_2_, WSe_2_, Cr_x_Se_y_, Graphene	110	500	-	4–31	0.2	**a-BN** film	2022 [104]
		Graphene/Ge, SiO_2_/Si	100	250	-	20.4	-	**a-BN** film	2023 [105]
		Cu/SiO_2_, Cu/SrTiO_3_, Cu/Sapphire, Cu/Quartz	85	1080	13.6	monolayer	0.15	Single crystalline **h-BN** film	2023 [100]
	B_2_H_6_, NH_3_	Sapphire	-	1100–1300	24.6	3	0.05	**h-BN** film	2021 [51]
	BCl_3_, NH_3_	Sapphire	-	1000–1400	-	0.8 μm	10.83	**h-BN** film	2016 [101]
			-	1200	-	1.3 μm	10.83		2021 [102]
		Si (100)	-	900–1300	60–30	2.3 μm	38.33		2021 [103]
**APCVD**	AB	SiO_2_/Si	90	1360	25	50–160	1.17	**t-BN**||**h-BN**||transition layer|SiO_2_/Si	2023 [107]
	B_2_O_3_, (NH_4_)_2_CO_3_	Sapphire	B_2_O_3_: 450 (NH_4_)_2_CO_3_: 60	1000–1050	23.2	5.67–22.82	0.32	**h-BN** film	2025 [52]

In this table, T_dec_ refers to the decomposition temperature of solid B/N precusors. Unless otherwise specified, ‘sapphire’ in this table denotes c-plane sapphire.

### 3.4. h-BN Growth via APCVD

**Figure 5:**
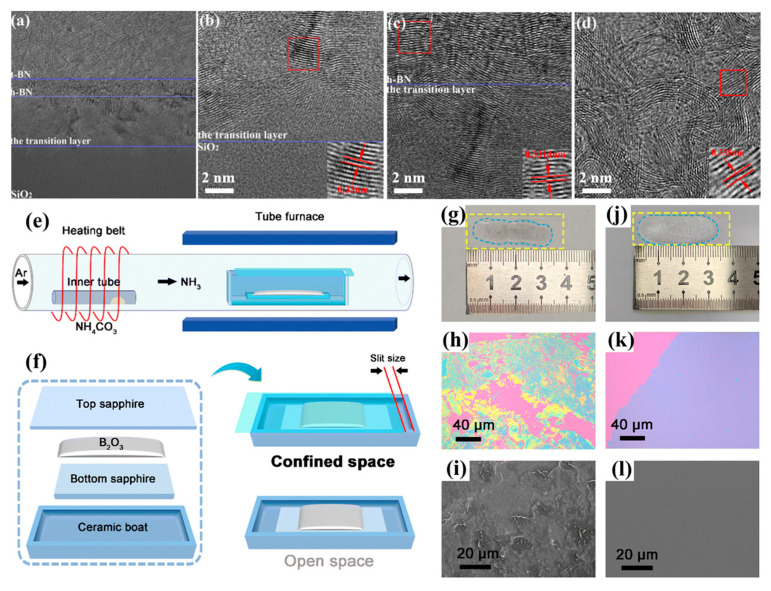
(**a**–**d**) The overall structure (**a**), the transition layer near the substrate (**b**), h-BN layer (**c**) and t-BN layer (**d**) of the cross-sectional HRTEM images of the h-BN film grown on SiO_2_/Si via APCVD [107]. (**e**,**f**) Cartoon of the APCVD experimental setup (**e**) and schematic contrast between confined space and open space methods (**f**) [52]. (**g**–**l**) Multilayer thin h-BN films grown in the open space (**g**–**i**) and confined space (**j**–**l**), respectively [52]. (**a**–**d**) Reproduced with permission [107]. Copyright 2023, IOP Publishing. (**e**–**l**) Reproduced with permission [52]. Copyright 2025, Royal Society of Chemistry.

Although most studies focus on low-pressure environments, some have explored the growth of h-BN under atmospheric pressure. In 2023, Yang used AB as a solid B/N source and N_2_ as the carrier gas to grow h-BN at 1360 °C [107]. HRTEM results showed that there is a transition layer with some ordered structures generated first on the SiO_2_/Si substrate, followed by the completely ordered h-BN layer with varying thicknesses from 10 to 50 nm (Figure 5a–d). With an increase in film thickness, t-BN started to form on top of the h-BN layer.

In 2025, Zhao et al. employed a dual solid-state precursor system comprising B_2_O_3_ and (NH_4_)_2_CO_3_ to synthesize h-BN films on sapphire [52]. The NH_3_ concentration was regulated via thermal decomposition of (NH_4_)_2_CO_3_ in a dedicated tube (See Figure 5e) via (NH_4_)_2_CO_3_ → 2NH_3_↑ + CO_2_↑ + H_2_O↑. The growth process was spatially confined between two sapphire wafers within an APCVD system (Figure 5f), enabling reduced NH_3_ partial pressure coupled with elevated B_2_O_3_ vapor concentration to synergistically suppress growth kinetics, achieving precise thickness control and uniform surface morphology (Figure 5g–l). The APCVD growth results have also been compiled into Table 3.

### 3.5. h-BN Growth via PECVD

PECVD has become a promising method for growing h-BN, primarily due to its low growth temperature, making it compatible with temperature-sensitive substrates and semiconductor devices. The deposition systems used are primarily radio frequency plasma-enhanced chemical vapor deposition (RF-PECVD), microwave-substrate wave plasma chemical vapor deposition (MPCVD), and inductively coupled plasma chemical vapor deposition (ICP-CVD). By applying different frequencies to excite gases into plasma, high-energy species within the plasma initiate reactions with precursor molecules, while plasma-activated dissociation pathways enable precise control over h-BN nucleation kinetics. This technique achieves h-BN crystallization at temperatures substantially below those required for conventional CVD.

Table 4 summarizes the key results of h-BN growth on insulating substrates achieved via PECVD [48,49,108,109,110,111,112,113,114,115,116]. Early research on PECVD mainly focused on using B_2_H_6_, and a small amount of organoboron precursors such as TMOB and DMAB, together with N_2_ or NH_3_, for the deposition of h-BN. In 1997, Carreño et al. pioneered one of the earliest studies using B_2_H_6_ and N_2_ as B/N sources [108]. They successfully deposited BN films of varying thicknesses (30–120 nm) onto polysilicon at temperatures ranging from 200 to 500 °C, carefully adjusting the gas flow rates and RF power. The resulting films contained a complex mixture of h-BN, c-BN, and a-BN. Notably, the phase composition was significantly influenced by both the RF power density and the N_2_/B_2_H_6_ flow ratio, with higher power densities and flow ratios resulted in a decreased presence of h-BN. In 2000 and 2002, Vilcarromero et al. synthesized h-BN on crystalline silicon (c-Si) via RF-PECVD at temperatures lower than 400 °C using B_2_H_6_/N_2_, and their samples also exhibited mixed-phase compositions containing h-BN, c-BN, and a-BN [109,110]. Therefore, significant challenges remain in achieving phase-pure h-BN below 500 °C using B_2_H_6_ precursors in PECVD systems, as boron nitride polymorph selectivity becomes highly sensitive to incomplete precursor dissociation kinetics and competing gas-phase reactions under low thermal budgets.

In addition to inorganic boron sources, some studies have utilized organic boron precursors. In 2001, El-Yadouni et al. examined the anisotropy of polycrystalline h-BN films synthesized using DMAB /N_2_ and measured their electro-optic coefficients [49]. In the same year, Thamm et al. successfully grew h-BN films on Si (100) using MPCVD with TMOB/N_2_ as the precursors [48]. They noted that substrate temperature significantly impacts the stability of the deposited layer. At lower temperatures, the film is more likely to decompose when exposed to humidity, while at higher temperatures, more stable h-BN films can be obtained. However, the contamination of h-BN samples with carbon impurities from organic precursors remains a challenge, resulting in multiphase mixtures and necessitating improvements in structural purity.

**Figure 6:**
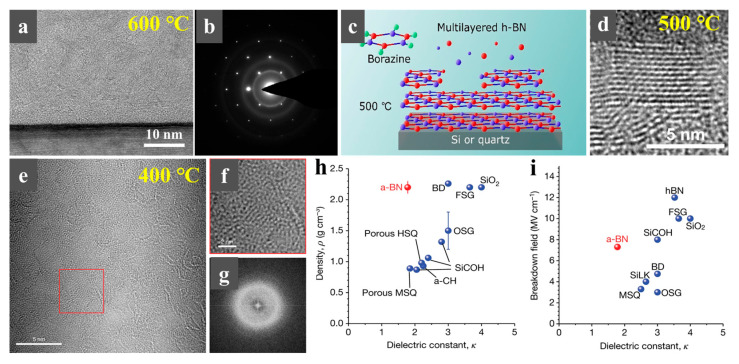
(**a**,**b**) High-resolution cross-sectional TEM images of h-BN (**a**) and the selected-area electron diffraction pattern of h-BN film grown at 600 °C via RF-PECVD (**b**) [112]. (**c**,**d**) Schematic diagram of h-BN grown on Si or quartz at 500 °C by ICP-CVD (**c**) and the corresponding HRTEM image of h-BN film (**d**) [114]. (**e**) HRTEM image of a-BN grown on Si at 400 °C via ICP-CVD. (**f**,**g**) Magnification of the area indicated by the red box in (**e**) and the Fast Fourier transform results for the area depicted in (**f**), demonstrating a diffuse diffraction pattern that is typical of an amorphous film (**g**) [113]. (**h**) Density versus dielectric constant for low-κ materials reported in the literature (blue circles) and a-BN (red circle) [113]. (**i**) Breakdown field versus dielectric constant for low-κ materials reported in the literature (blue circles) and for a-BN (red circle) [113]. (**a**,**b**) Reproduced with permission [112]. Copyright 2015, Springer Nature. (**c**,**d**) Reproduced with permission [114]. Copyright 2023, ACS. Reproduced with permission [113]. Copyright 2020, Springer Nature.

Recently, advancements demonstrate a precursor engineering strategy in the PECVD synthesis of h-BN, where researchers bypass conventional dual-source (such as B_2_H_6_ + N_2_/NH_3_) configurations through single-source precursors like borazine [112,113,114]. In 2015, Merenkov et al. first synthesized Borazine through the reaction of ammonium chloride (NH_4_Cl) and lithium tetrahydridoborate (LiBH_4_), then successfully grew h-BN nanowalls on Si (100) substrates with a borazine + ammonia mixture (Figure 6a,b). The experiments determined the conditions for forming a-BN at 300 °C and nanocrystalline h-BN at 600 °C [112]. In 2023, Yamamoto et al. further explored the deposition of multilayer h-BN directly onto silicon wafers at 500 °C using borazine via ICP-CVD [114]. They elucidated the roles of N_2_/H_2_ carrier gases in h-BN growth, demonstrating that their introduction not only critically modulates deposition kinetics but also enables enhanced crystallinity of h-BN (Figure 6c,d). In PECVD, borazine compounds undergo plasma-mediated fragmentation, enabling in situ generation of activated boron and nitrogen species without requiring external N_2_/NH_3_ gas streams. In 2020, Hong et al. fabricated 3 nm thick a-BN using borazine as the single-source precursor via ICP-CVD at 400 °C (Figure 6e–i) [113]. The dielectric constants of the film were measured at 1.78 and 1.16 at operating frequencies of 100 kHz and 1 MHz, respectively, which are close to the dielectric constant of air (κ = 1). This represents one of the lowest known dielectric constants to date, significantly below the International Roadmap for Devices and Systems (IRDS) 2028 target of κ < 2 [113].

Another precursor type, the solid-state single B/N source AB, has also been utilized for the PECVD growth of h-BN [115,116]. AB undergoes thermal sublimation with its vapor entrained in a carrier gas stream and delivered into the plasma zone, where dissociation generates plasma-activated boron/nitrogen radicals. These species subsequently undergo surface-mediated recombination to grow h-BN films. In 2019, Singh et al. demonstrated low-temperature (500 °C) synthesis of polycrystalline h-BN on a Si substrate using an AB precursor via MPCVD [115]. However, an XPS study revealed residual carbon contamination and oxygen incorporation, attributed to incomplete precursor decomposition and ambient-induced oxidation after growth. In the same year, Liu et al. reported the growth of uniform polycrystalline h-BN with an AB precursor and Ar/H_2_ carrier gas via RF-PECVD on various substrates, including SiO_2_/Si, quartz, sapphire, and silicon at 300–500 °C. By controlling the growth time, the film thickness was reported to be adjusted from a monolayer to four layers [116].

**Table 4:** Growth of h-BN films on insulating substrates via PECVD.

Technique	B Precursor	N Precursor	Substrate	Growth Temperature (°C)	Thickness (nm)	Deposition Rate (nm/min)	Structure	Year
RF-PECVD	B_2_H_6_	N_2_	Polysilicon	200–500	30–120	3	Mixture of **h-BN**, **c-BN** and **a-BN**	1997 [108]
RF-PECVD			c-Si	400	-	0.05–2	Mixture of **h-BN**, **c-BN** and **a-BN**	2000 [109]
RF-PECVD			c-Si	180, 340	-	0.1–2	Mixture of **h-BN** and **a-BN**	2002 [110]
MPCVD	TMOB	N_2_	Si (100)	650–800	800 °C: 300	2.5	**h-BN** film	2001 [48]
MPCVD	DMAB	N_2_	Galss-ITO	350	-	-	**h-BN** film	2001 [49]
PECVD		-	Si, Quartz	280–550	0.1 μm–1 μm	-	Mixture of **h-BN** and **c-BN**	2005 [41]
RF-PECVD	Borazine	NH_3_	Si (100)	100–700	30–400	10–22	100–200 °C: **a-BN** film 300–700 °C: **h-BN** nanowalls	2015 [112]
ICP-CVD		-	Si	400	3	0.03	**a-BN**	2020 [113]
ICP-CVD		N_2_	Si, Quartz	500	10	1.8	**h-BN** film	2023 [114]
MPCVD	AB	-	Si	500	12	4	**h-BN** film	2019 [115]
RF-PECVD		-	Sapphire, Si, Quartz, SiO_2_/Si	300–500	0.85–2.1	0.03	**h-BN** film	2019 [116]

## 4. Summary and Future Perspectives

The development of CVD techniques for growing h-BN on insulating substrates has advanced significantly, offering alternatives to metal-catalyzed growth and avoiding transfer-related defects. This review systematically examines the synthesis of h-BN on insulating substrates via three principal CVD techniques: MOCVD, LPCVD, and PECVD, with APCVD showing limited applicability.

Growth temperature plays a critical role in determining h-BN crystalline quality. While MOCVD and LPCVD require elevated temperatures (>1100 °C) for polycrystalline h-BN growth and even higher ranges (1300–1400 °C) for single-crystalline formation, PECVD enables polycrystalline deposition at significantly reduced temperatures (300–800 °C). All three techniques demonstrate excellent thickness controllability, spanning monolayer/bilayer configurations to micron-scale films, highlighting their versatility in dimensional control.

The growth rates of h-BN exhibit significant variations across different CVD techniques. Notably, MOCVD-grown h-BN shows the widest range, spanning two orders of magnitude from 0.05 to 100 nm/min. Comparatively, LPCVD exhibits growth rates be-tween 0.1 and 38 nm/min, while PECVD demonstrates a range of 0.05 to 22 nm/min. Critically, MOCVD demonstrates the broadest tunability—enabling both precise atomic-layer control and rapid growth of thick samples.

The selection of B/N precursors fundamentally determines the crystallographic phase and quality of synthesized BN materials. In MOCVD systems, the conventional TEB/NH_3_ precursor combination facilitates h-BN growth but exhibits limited phase control when adapted to LPCVD, where it predominantly yields t-BN or r-BN phases due to altered decomposition kinetics. LPCVD predominantly utilizes AB as a self-contained source, achieving superior h-BN phase purity through controlled thermal cleavage of the B-N bond. PECVD demonstrates broader B-source compatibility (excluding B_2_H_6_, which induces mixed h-BN/c-BN/a-BN phases) but requires separate nitrogen precursors. Recent advances reveal innovative PECVD configurations utilizing AB as a single-source precursor, eliminating external nitrogen inputs. The observed technique-dependent precursor reactivity underscores the importance of process-specific optimization, as identical precursors manifest distinct growth mechanisms and phase outcomes across different CVD platforms. Such findings mandate platform-tailored optimization protocols to advance h-BN synthesis toward tailored crystallographic and functional properties.

Despite significant advances in h-BN synthesis on insulating substrates, critical challenges persist that demand innovative solutions. Foremost among these is the prohibitively high growth temperature required for crystalline growth of h-BN. Current polycrystalline h-BN deposition typically exceeds 1100 °C, while single-crystal synthesis necessitates extreme temperatures (>1300 °C). This thermal mismatch fundamentally limits h-BN’s adoption in hybrid heterostructures and temperature-sensitive semiconductor integration schemes. Emerging strategies such as plasma-catalytic LPCVD and metastable precursor engineering show promise in reducing growth temperatures below 800 °C without compromising crystallinity. The continued advancement of low-temperature deposition techniques promises to significantly expand the application horizons of boron nitride. PECVD has achieved a-BN growth below 200 °C while enabling polycrystalline h-BN formation above 300 °C. LPCVD has demonstrated controllable a-BN preparation under 300 °C. These developments create new possibilities for integrating BN with temperature-sensitive substrates—particularly organic and polymeric materials that have traditionally been incompatible with conventional high-temperature processes.

A second critical challenge involves achieving crystalline quality comparable to metal-catalyzed growth. The Raman FWHM of h-BN grown by LPCVD on sapphire ranges from 25 to 40 cm^−1^, while that of h-BN grown at 300 °C with copper assistance ranges from 14 to 20 cm^−1^ [12,13,117,118,119]. This significant discrepancy highlights the urgent need for innovative substrate engineering approaches, including atomic-scale surface reconstruction, strain-tuned buffer layers, and chiral-selective nucleation templates to narrow the quality gap.

Doping engineering remains underdeveloped but essential for tailoring h-BN’s electronic properties. While h-BN’s wide bandgap and chemical inertness complicate conventional impurity incorporation, the development of in situ doping protocols could unlock tailored electronic/optical functionalities. Machine learning-driven precursor optimization could achieve precise dopant species/concentration control, advancing h-BN-based quantum emitters and correlated electron devices.

The precise thickness control of h-BN is critical for multiscale applications. Quantum photonic applications demand atomic-level uniformity, whereas dielectric applications typically need continuous films ranging from tens of nanometers to micrometers. The Precise thickness control of high-quality h-BN on insulating substrates remains a critical challenge, requiring breakthroughs in nucleation regulation and growth kinetics modulation.

Environmental durability emerges as a critical yet nascent research direction for insulating substrate-grown h-BN. Emerging evidence confirms superior long-term stability in insulating substrate-supported h-BN compared to metal-supported counterparts, primarily attributed to oxygen intercalation at metal/h-BN interfaces under ambient conditions [96]. This fundamental insight positions insulating substrate-grown h-BN as a transformative platform for stability applications—including high-temperature operation, oxidation resistance, and corrosion mitigation—making it a compelling frontier for next-generation durable device architectures.

Addressing these scientific and technical barriers will be essential for realizing the full potential of h-BN for next-generation electronics, optoelectronics, and advanced thermal management systems.

## Data Availability

No new data were created or analyzed in this study.
